# Convolutional Neural Networks for Classification of T2DM Cognitive Impairment Based on Whole Brain Structural Features

**DOI:** 10.3389/fnins.2022.926486

**Published:** 2022-07-19

**Authors:** Xin Tan, Jinjian Wu, Xiaomeng Ma, Shangyu Kang, Xiaomei Yue, Yawen Rao, Yifan Li, Haoming Huang, Yuna Chen, Wenjiao Lyu, Chunhong Qin, Mingrui Li, Yue Feng, Yi Liang, Shijun Qiu

**Affiliations:** ^1^First Clinical Medical College, Guangzhou University of Chinese Medicine, Guangzhou, China; ^2^Medical Imaging Center, First Affiliated Hospital of Guangzhou University of Chinese Medicine, Guangzhou, China

**Keywords:** type 2 diabetes mellitus, cognitive impairment, classification, convolutional neural networks, MRI

## Abstract

**Purpose:**

Cognitive impairment is generally found in individuals with type 2 diabetes mellitus (T2DM). Although they may not have visible symptoms of cognitive impairment in the early stages of the disorder, they are considered to be at high risk. Therefore, the classification of these patients is important for preventing the progression of cognitive impairment.

**Methods:**

In this study, a convolutional neural network was used to construct a model for classifying 107 T2DM patients with and without cognitive impairment based on T1-weighted structural MRI. The Montreal cognitive assessment score served as an index of the cognitive status of the patients.

**Results:**

The classifier could identify T2DM-related cognitive decline with a classification accuracy of 84.85% and achieved an area under the curve of 92.65%.

**Conclusions:**

The model can help clinicians analyze and predict cognitive impairment in patients and enable early treatment.

## Introduction

Type 2 diabetes mellitus (T2DM) can disrupt the balance that the human brain constructs for regulating blood glucose levels, which causes insulin resistance and elevated blood glucose levels. This may result in an imbalance of energy supply in the brain tissue, which can lead to irreversible damage over long term. During this process, some patients with T2DM may experience various cognitive impairments (Srikanth et al., 2020; You et al., 2021), including memory, executive ability, and affective disorders; T2DM is associated with a higher risk of dementia. Previous studies have shown that T2DM is one of the main risk factors for Alzheimer's disease (AD) (Biessels and Despa, 2018). Some patients with T2DM do not present cognitive impairment (T2DM-noCI); however, they belong to high-risk groups. This stage is the best intervention period to prevent the progression of cognitive impairment. Therefore, a classification model is important for clinicians to provide early treatment.

Previous neuroimaging studies established that extensive structural damage occurred in patients with T2DM. Geijselaers et al. (2015) confirmed that cognitive impairment in T2DM is associated with impaired brain structure and is closely related to the stability of blood glucose levels and insulin resistance. Espeland et al. (2013) conducted a 3–6-year follow-up study on patients with T2DM, and they established that the probability of gray matter volume decreasing gradually increased with time, and the cortex gradually became thinner. Vergoossen et al. (2020) demonstrated abnormal changes in the structure of the white matter in patients with T2DM, and these abnormalities were correlated with cognitive impairments. Espeland et al. (2016) established that patients with T2DM had increased white matter hyperintensities and decreased brain tissue volume. However, the adverse effects of the brain structure could be reduced after controlling body weight. Erus et al. (2015) revealed that lower gray matter volumes are associated with long-term T2DM, and intensive glycemic treatment could slow down this process. Yao et al. (2021) established that the gray matter volume decreased in the limbic system. Lee et al. (2018) proved that the brain volume of patients with T2DM decreased to different degrees in subjects with mild cognitive impairment and dementia, which was closely related to the decrease in cognitive function. Sanjari Moghaddam et al. (2019) established that the white matter microstructure of some brain regions, such as the frontal lobe, temporal lobe, parietal lobe, and cingulate gyrus, was damaged, which was closely related to the decrease in memory and attention, information processing ability, as well as executive ability. Based on the results of previous studies on the structural abnormalities of the brain in patients with T2DM, we used high-resolution three-dimensional T1-weighted imaging (3D-T1) to obtain brain structural images of patients and used them for image recognition training of the classification model.

In recent years, the use of machine learning methods, such as support vector machines (SVMs), k-nearest neighbors, random forests, and other ensemble classifiers, to analyze MRI images to predict the disease stage of patients has achieved good results (Segar et al., 2019; Aminian et al., 2020; Gou et al., 2021; Huang et al., 2021). However, these conventional machine learning methods have significant limitations (Yamanakkanavar et al., 2020). Before analyzing the features extracted from the brain regions and predicting the results of patient status, it is necessary for the machine learning methods to select the brain regions with significant abnormalities manually. Artificial extractions are based on existing clinical or experimental experience, and they might overlook some useful areas that have not been found at present. Moreover, when the number of layers of the neural network is increased, the conventional network encounters problems such as local optimization, overfitting, and gradient diffusion.

Deep-learning technology is a branch of machine learning that imitates the thinking of the human brain. It applies a back-propagation learning algorithm to learn the parameters of a deep neural network (Carin and Pencina, 2018) and has the ability to represent learning and input information filtering layer-by-layer, which can realize end-to-end supervised and unsupervised learning. A convolutional neural network (CNN) is a deep neural network with a convolution structure inspired by the receptive field mechanism in biology (Qiu et al., 2018, 2020; Wen et al., 2020). This is a deep learning model for image processing. The CNN has two advantages over other machine learning methods. First, in addition to the full connection layer and the output layer, the neurons in the CNN use partial connections, whereas those in the conventional neural networks are all connected. Second, the CNN shares weights among the neurons in the same layer to reduce the complexity of the network model. These characteristics make the CNN more suitable for image feature learning and expression, as compared to other deep learning methods. To date, this model has been used for every major breakthrough in the field of image recognition. The CNN and many models derived from it have been used in classification tasks of cognitive impairments and they have achieved excellent results. Therefore, we used the CNN to construct a classification model for cognitive impairment in patients with T2DM.

## Research Design and Methods

### Participants

In this study, 107 participants (45 T2DM patients with cognitive impairment and 62 without) from the Endocrine Department in the first affiliated hospital of Guangzhou University of Traditional Chinese Medicine were enrolled. T2DM was defined according to the latest criteria published by the American Diabetes Association: HbA1c ≥ 6.5% (48 mmol/mol); fasting blood glucose ≥ 7.0 mmol/L (126 mg/dL); oral glucose tolerance test 2 h postprandial blood glucose ≥11.1 mmol/L (200 mg/dL); symptoms of hyperglycemia or hyperglycemic crises; and random blood glucose≥11.1 mmol/L (200 mg/dL), without symptoms of hyperglycemia, and the standard of 1 to 3 items was reviewed. Participants were excluded if they had a history of psychiatric diseases; stroke; epilepsy; head trauma; brain surgery; cerebrovascular accidents; obvious cognitive impairment who find it difficult to cooperate with the cognitive scale test; or had severe liver, kidney, or heart disease (like coronary heart disease, heart failure); and rheumatoid- and thyroid-related diseases (especially hyperthyroidism). A transient ischemia attack in the past 2 years, alcohol or tobacco abuse, severe hypertension (systolic pressure ≥ 160 mmHg or systolic pressure ≥ 110 mmHg), and contraindications to MRI were also exclusion criteria, as were specific brain abnormalities on conventional MR scans. Moreover, the patients with T2DM were excluded if they had unstable blood glucose control, acute or chronic metabolic complications of clinical diabetes and severe hypoglycemia, or a history of ketoacidosis.

### Medical History and Biometric Measurements

Medical history and medication use were recorded with a standardized questionnaire, and patients in this study were mainly treated with insulin and metformin. Systolic and diastolic blood pressure, biometric examinations, and body mass index (BMI) were measured. The biometric examinations, including those of averaged fasting glucose, HbA1c, fasting C-peptide, total cholesterol, triglycerides, and low density lipoprotein were measured with standard laboratory testing.

### Data Augmentation

The sample size for the training dataset was 74. To design a better model and improve the generalization ability of the model, we used 3D random rotation for data augmentation. The steps are as follows. First, MR images were normalized *via* nonlinear transformation and picture size adjustment. Second, we obtained the center coordinates of rotation, that is, the center coordinates of the image. Third, the rotation matrix, rotation box, rotation landmarks, and image rotation were achieved. Finally, the sample number was amplified 10 times that of the original data.

### Cognitive Assessment

All participants underwent a series of neuropsychological tests that evaluated general cognitive function, memory, attention, executive function, and visuospatial skills, including the Montreal Cognitive Assessment (MoCA, Beijing edition), the auditory verbal learning test, the trail-making test, Grooved pegboard test, digit-symbol test, and digital sequence test. MoCA scores served as an index of the cognitive status, which divided our T2DM patients into two groups, T2DM patients with cognitive impairment (T2DM-CI) group and T2DM-noCI group. Participants whose MoCA score was less than 26 were considered to have cognitive impairment. The AVLT contains three parts, including immediate tasks, 5-min tasks, and 20-min delayed recall tasks, which were used to assess short-term and delayed memory, and the digital sequence test was used as a simple method to assess immediate memory. The trail-making tests were mainly used to evaluate attention and psychomotor speed. All the tests took approximately 30 min to administer.

### MRI Dataset Acquisition

MRI 3D-T1 was acquired as the brain structural images to classify T2DM patients with and without cognitive impairment. Model training, internal validation, and testing were performed on the dataset.

MR images were all acquired with a 3T Siemens Prisma with an 8-channel head coil. Conventional brain axial T1-weighted, T2-weighted, and FLAIR images were obtained for every subject to exclude organic disease. Subjects were instructed to keep their eyes closed but to remain awake and to keep their heads still during the scan. Head motion was controlled as much as possible using foam padding, and scanner noise was reduced using earplugs. Structural images were acquired with the following parameters: TR = 2,000 ms, TE = 2.6 ms, inversion time = 450 ms, flip angle = 12°, matrix = 256 × 256, field of view = 250 × 250 mm, and 256 continuous sagittal slices with 1-mm thickness. The voxel of 3D-T1 image in this study was 1 × 1 × 1mm.

### Brain Extraction

Before extracting the brain tissues, MRI images were first converted from DICOM format into ANALYZE in a recursive routine using the Python language. The brain extraction tool (BET) in the FMRIB Software Library was used to extract the brain tissue. The BET algorithm is a common brain tissue extraction method based on a deformable lattice model. The algorithm has high robustness and accuracy, and is computationally fast, making it a good method for extracting the brain tissue. This method first calculated the gray-scale image of MRI brain images and estimated three values, including the gray threshold, the maximum value, and the maximum value for distinguishing the brain and other tissues. Thereafter, the algorithm estimated the focus of the brain tissue and obtained the initial brain tissue according to the gray values. Finally, the initial brain surface was constructed using a 3D triangle facet. Tangential and smoothing forces were built in every triangle facet, which caused the initial brain surface to maintain a fixed distance and smoothness and made the brain surface sufficiently smooth and stable.

### Features Addition

We selected 21 features, including clinical data and neuropsychological test scores. The clinical data included HbA1c, fasting blood glucose, fasting insulin, systolic blood pressure, total cholesterol, triglycerides, low-density lipoprotein, fasting C-peptide, and BMI. Neuropsychological test scores included the auditory verbal learning test (immediate, 5 mins, delay, recall), the trail-making test, Grooved pegboard test, the Mini-Mental State Exam, digit-symbol test, and digital sequence test. The features above were then filled with null values and normalized to 0–1, and then merged with the 3D image as an additional layer.

### CNN Construction and Training

We constructed an 11-layer 3D CNN. The steps for constructing the network mainly included image input, convolution, rectified linear unit, pooling, and image classification. The network structures are listed in Table 1. In the table, the size of the input layer was 64 × 64 × 11, wherein 64 × 64 refers to the size of pixels, and 11 refers to the number of channels, which was obtained by 3D scaling of 256 layer images. Starting from the second row, the number in the second column represents that corresponding to the third column. For instance, 11 in the second row refers to 11 convolution layers. Subsequently, the model was trained. We input the images and evaluated the network after each loop, and training was stopped. The network was updated after 10 iterations with no loss improvement. The samples were normalized samples with mean 0 and var = 1 before being passed to the net. Randomized samples were selected from the dataset during training. We used a fold cross-validation to verify the stability of the model. In this model, the precision, accuracy, recall, F1 score, and area under the curve (AUC) were calculated. The formulae are as follows.


$$\begin{array}{l} \text{Precision}=\text{TP}/(\text{TP}+\text{FP})\\ \text{ Recall}=\text{TP}/(\text{TP}+\text{FN})\\ \text{Accuracy}=(\text{TP}+\text{TN})/(\text{TP}+\text{FP}+\text{TN}+\text{FN})\\ \text{ F1 score}=\text{P x R}/2(\text{P}+\text{R}) \end{array}$$

(TP: true positive, FP: false positive, TN:true negative, FN: false negative, P: precision, R: recall).

**Table 1:** The information on the 11-layer 3D CNN.

	**Number of layers**	**Layer category**
Layer 1	1	input
Layer 2	11	conv
Layer 3	11	batchNorm
Layer 4	17	conv
Layer 5	17	batchNorm
Layer 6	34	conv
Layer 7	34	batchNorm
Layer 8	1,024	fc
Layer 9	2	fc
Layer 10	2	softmax
Layer 11	2	output

*The input layer (Layer 1) size was 64 × 64 × 11*.

## Results

### Characteristics of Patients

A total of 107 participants were recruited for this study, included 45 T2DM-CI and 62 T2DM-noCI. The average age of all patients was 48.78 years, and 59.81% of patients were men (64 of 107). Moreover, the average MoCA score of the patients was 25.15 (Table 2). Brain structural MRI data of the 107 patients enrolled.

**Table 2:** Characteristics of included T2DM studies.

**Characteristics**	**T2DM patients (*n* = 107) (mean ±SD)**
Age (year)	48.78 ± 9.24
Sex (male/female)	64/43
Education level	10.27 ± 4.09
HbA1c % (mmol/mol)	9.34 ± 2.45
BMI (kg/m2)	24.23 ± 2.80
MoCA	25.15 ± 3.61

*SD, standard deviation; HbA1c, glycated hemoglobin; BMI, body mass index; MoCA, Montreal Cognitive Assessment*.

### Deep Learning Network Training and Performance

**Figure 1:**
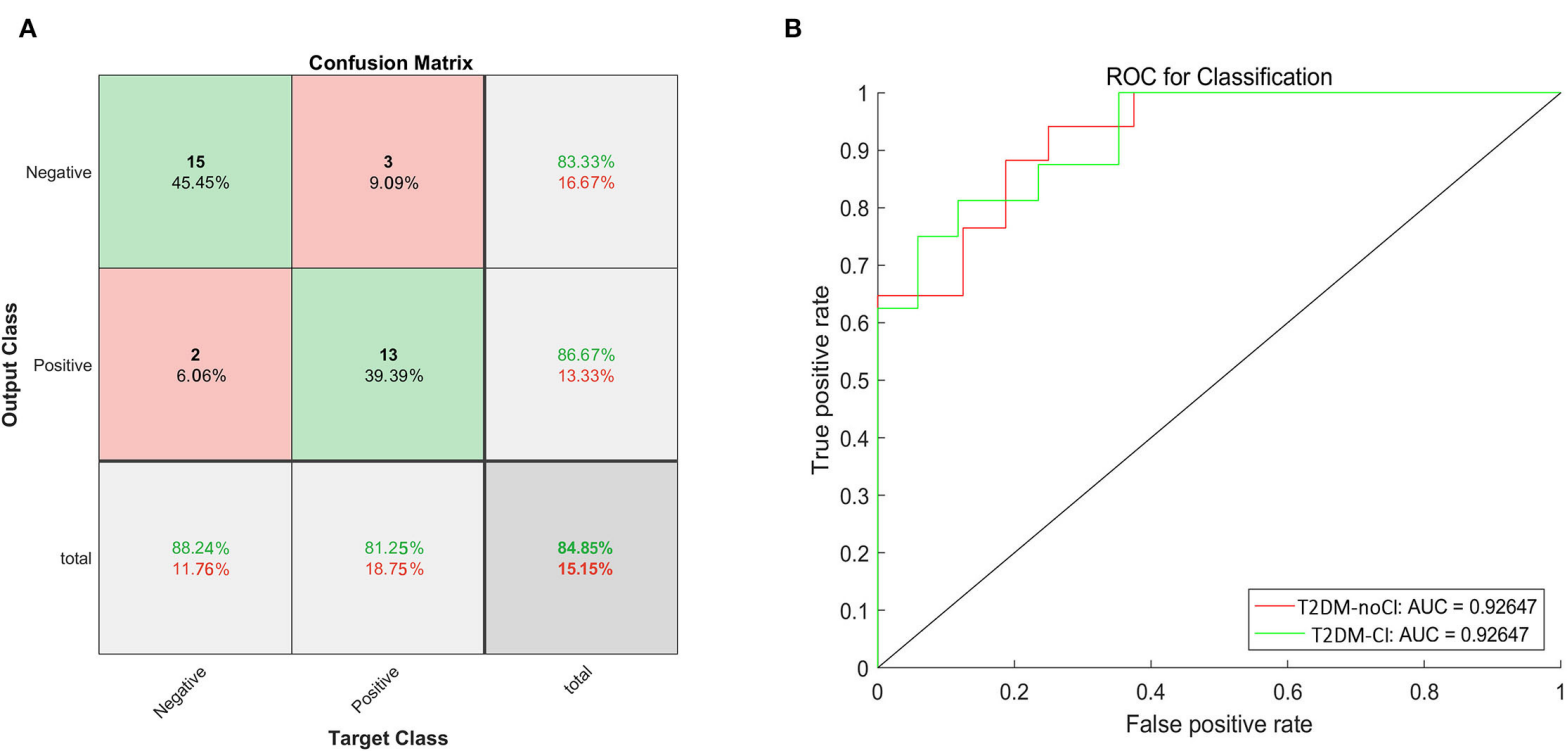
The confusion matrix and the ROC curve of the test dataset. **(A)** The green boxes represent the true negative and positive rates, and the red boxes represent the false negative and positive rates. The dark gray boxes in the lower right corner represent the accuracy. The accuracy was 84.85%. **(B)** The ROC curve to classify cognition, the red line represented the ROC of T2DM-noCI, and the green line represented the ROC of T2DM-CI. The AUC was 92.65%.

In this study, 74 samples were the training set and 33 samples were designed as the test set. The training set and the test set were randomly assigned. The training set included 42 T2DM patients with cognitive impairments and 32 without. The test set included 15 T2DM patients with cognitive impairments and 18 without. Initially, only 3D-T1 images were used to construct the model. The accuracy of the model was 75.80 % and it achieved an AUC of 74.27%. After adding 21 clinical features, the accuracy of the model was 84.85% (Figure 1A) and it achieved an AUC of 92.65% (Figure 1B). On the other hand, the precision of this model was 86.67%, the recall was 81.25%, and F1 score was 83.87%. Ten-fold cross validation was done and its AUC was 0.79231.

**Figure 2:**
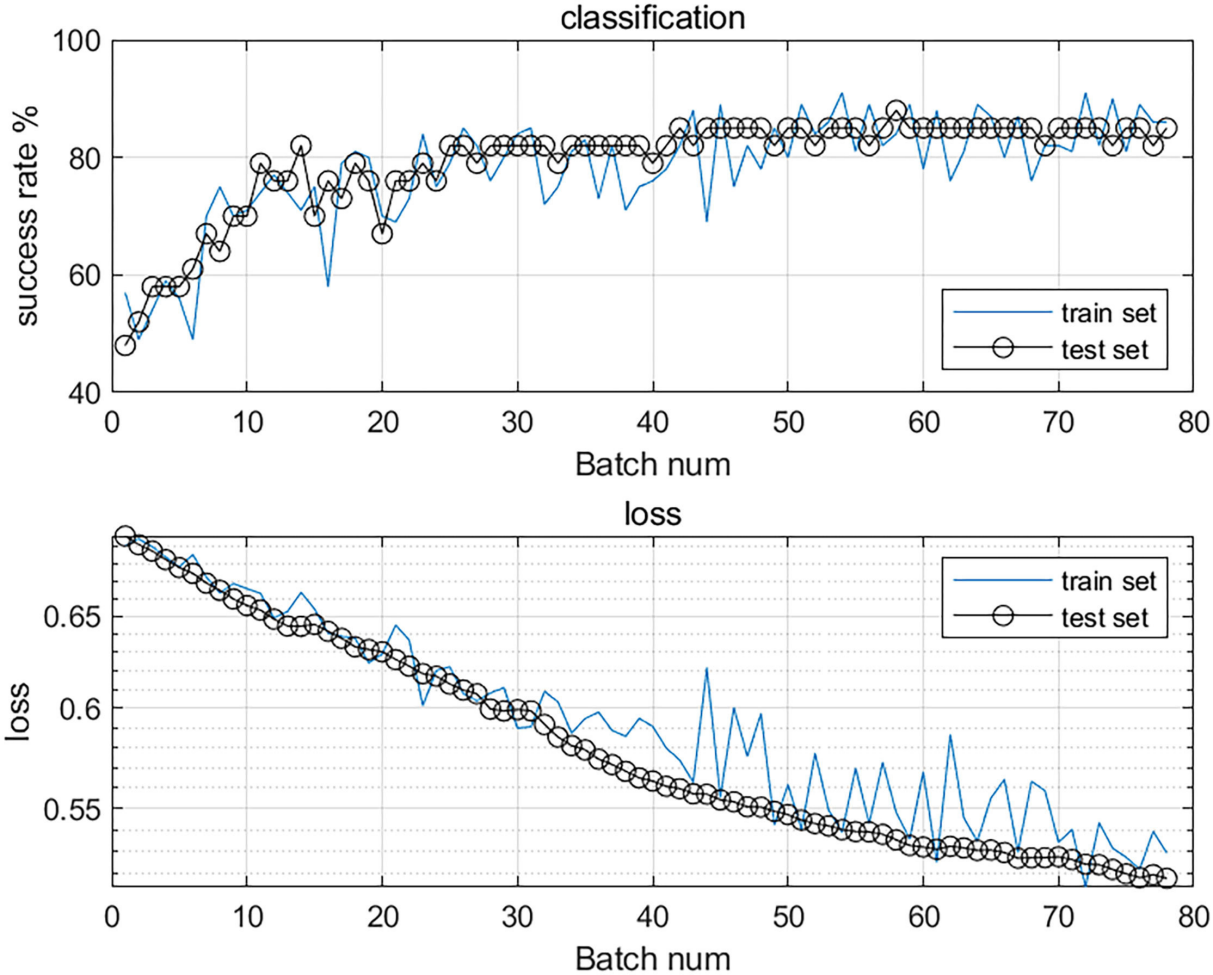
The accuracy and loss value curves of the classification model. The top curve shows the overall success rate, the increasing success rate proves that its accuracy is improving. The bottom curve shows the loss curves of the training and test sets, the loss value is decreasing, which proves that the model is converging and has good stability.

After the final training, we obtained the training curve (Figure 2). The loss curves of the training dataset and the test dataset showed a downward trend, and the overall success rate of learning was on the rise.

## Discussion

In this study, we employed a deep learning method for the classification of T2DM patients with and without cognitive impairments based on MRI 3D-T1 brain structural images. After brain extraction, data augmentation, and selection of 21 features to help neural networks learn, CNN construction, and training, we constructed a model for classification. After testing the test set and performing model validation of the training set, the loss curves showed a downward trend, and the overall success rate of learning increased, which indicated that the model had good discrimination ability. The model achieved performance with an AUC of 0.9265. The accuracy of classification was 84.85%.

### The Clinical Significance of the Model

Previous studies have shown that greater cognitive decline occurs among patients with T2DM, as compared to the general nondiabetic population of the same age; therefore, T2DM patients may present cognitive disorders at an earlier age. However, although the incidence rate of T2DM individuals affected by cognitive dysfunction has gradually increased, sufficient clinical attention has not been paid to the development of cognitive impairments in patients with T2DM. Currently, some cognitive tests, such as the MoCA scale, are widely used in the clinical diagnosis of cognitive impairment. Many previous neuroimaging studies have used the MoCA scale for grouping. The MoCA scale can achieve relatively good accuracy for multiple samples. Recognizing its important role, this study used the MoCA scale for grouping. However, for individuals, it may produce some bias, which is closely related to the tester's test level, test environment, and subject's cooperation. Our model aims to use images as a more objective method to diagnose T2DM cognitive impairment. A scanned image can more objectively show pathological characteristics and significantly reduce human bias than manual classification using a scale.

Since the cause of AD is irreversible (Yang et al., 2021) and there is no cure, the early diagnosis of AD is crucial for treatments to slow down the development of the disease. Various studies have confirmed that T2DM is one of the main risk factors for AD, and patients may experience various cognitive impairments, such as memory loss, executive ability reduction, and affective disorder. In contrast to the cognitive impairments in AD, most of the cognitive dysfunctions in patients with T2DM are reversible. Specifically, the best stage to implement preventive measures is when T2DM patients present no cognitive impairment. However, in the research of classification and prediction models of cognitive impairments, most of them focus on AD, specifically the evolution and prediction of mild cognitive impairment (MCI) in AD. Few studies have focused on T2DM-related cognitive disorders. Lin et al. (2018) studied and classified the features of brain MRI images in patients with MCI and AD using multi-dimensional MRI information to establish an AD prediction model. Zhu et al. (2021) proposed a temporally structured SVM model to classify MCI and AD to achieve early detection and diagnosis. Hett et al. (2021) presented a novel graph-based grading framework to combine the features and a multiscale approach that enabled the analysis of alterations of whole-brain structures and classified MCI and AD with an AUC of 85%. Jin et al. (2020) used a deep learning model (3D attention network, 3DAN) to classify MCI and AD based on structural MRI; the model demonstrated good performance in predicting MCI subjects who will progress to AD with an accuracy of 72%. Jie et al. (2020) designed a wc-kernel-based CNN framework for learning the features of MCI and AD diagnosis using fMRI. These previous studies on cognitive impairment in AD can provide a methodological and theoretical basis for this study.

In this study, we combined imaging and clinical objective information and built a classification model using the CNN, which achieved performance with an AUC of 0.9265. The accuracy of classification was 84.85%. These results indicated that the model has good discrimination ability in classifying T2DM patients with and without cognitive impairments. It can help clinicians objectively analyze and predict the cognitive impairment of patients by combining imaging and clinical data, not by using neurological test scales alone, which may cause human error. In addition, according to the classification results, clinicians can treat patients at an early stage, as it is important to prevent the development of dementia and improve the quality of life of patients.

### The Superiority of the Model

Before constructing the final model, we attempted to use 3D-T1 images only to construct the model. The accuracy rate reached 75.80%. As the sample size was not sufficiently large, the accuracy rate did not attain our expected goal; therefore, we added 21 clinical features to increase the stability of the model. Further research will expand the sample size, increase the accuracy of image diagnosis, and achieve an ideal classification effect. In day-to-day clinical work, doctors can make diagnoses using scanned images. The 21 clinical features included HbA1c, fasting blood glucose, fasting insulin, and systolic blood pressure. The selection of these clinical features was based on the degree to which they may affect T2DM or are closely related to the patient's cognition. For instance, the previous literature and our previous studies (Qin et al., 2019; Tan et al., 2019; Li et al., 2020) have confirmed that HbA1c is closely related to cognitive impairment in patients with T2DM. The higher the level of HbA1c, the more serious is the cognitive impairment of patients. In addition, cholesterol and triglycerides were confirmed to be related to a reduction in cognitive levels (Hakala et al., 2021). We did not consider diabetes duration as a clinical feature. As type 2 diabetes is a chronic disease with latent onset, we could not determine the exact onset time of disease in the patients. The onset time could be the time since the onset of the disease but not the real illness time. Moreover, the time of onset of type 2 diabetes can include the early asymptomatic stage. We believe that a sufficiently accurate estimation of the duration of diabetes cannot be achieved; therefore, we did not apply this index to the prediction model.

Few studies have been conducted on the classification of cognitive impairment in patients with T2DM. Most of these studies used conventional machine learning methods, such as the SVM classifier. Liu et al. (2019) marked whole-brain resting state functional connections (RSFCs) and used SVM to identify cognitive dysfunction in patients with T2DM. The SVM is one of the most common machine learning models. It can effectively distinguish features of different categories by constructing high-dimensional decision planes in feature space. It has an excellent performance in the case of small sample sizes and high dimensions; however, some of its disadvantages cannot be ignored. The extraction of image features is typically based on prior assumptions. Abnormal changes caused by T2DM might be overlooked because they are not considered in the assumptions, and some brain regions related to T2DM might not match the defined brain regions, resulting in a decline in the expression of the extracted regional features. Conversely, the features extracted by conventional machine learning methods rely on image preprocessing, such as strict registration and segmentation, and they need to go through various processing steps based on image denoising and normalization, which usually requires the guidance of experts in the field of brain science.

Deep learning is good at mining abstract-distributed feature representations from the original input data, and these representations have good generalization ability. Deep neural networks are the main forms at present, and CNN is one of the classic and widely used network structures. The CNN can effectively reduce the complexity of the network; reduce the number of training parameters; and make the model invariant to translation, distortion, as well as scaling. Thus, it has strong robustness as well as fault tolerance. Additionally, it is easy to train and optimize the network structure. It has significant advantages over shallow models in terms of feature extraction and model fitting. In this study, we used a 11-layer CNN to perform an automatic analysis of the whole-brain structure of patients with T2DM without extracting the relevant image features in advance, which reduced the experience difference. Moreover, we appropriately increased the depth of the network and added convolution layers, which extracted abstract features and improved the accuracy of the classifier. Finally, a classification model of cognitive impairment in T2DM patients was constructed, and a good classification effect was achieved.

Our T2DM cognitive impairment classifier was a 3D-CNN model. 3D-CNN achieves the automatic classification of diagnostic images by capturing the anatomical shape changes of MRI on strictly registered MRI data, combining three 3D convolution layers with a two-layer fully connected network for global fine-tuning training (Dolz and Desrosiers, 2017). Most relative classification models used in previous studies were based on the 2D-CNN method. However, when using 2D images to study 3D neural images, the information between the 3D structures in the images is usually ignored. The 3D-CNN is more accurate in the feature extraction of spatial information, which means that the network has higher accuracy (Singh, 2020; Singh et al., 2020). In our CNN model, the features of the voxel model were extracted using convolution layers; the extracted features were integrated by pooling layers; and finally, the model was classified in the full connection layers.

## Conclusion

In this study, we constructed a 3D 11-layer CNN model to classify T2DM patients with and without cognitive impairments. This model achieved performance with an AUC of 0.9265 and the accuracy of classification was 84.85%, which prompted to achieve a good classification effect. It can help clinicians objectively analyze and predict patients' cognitive impairment and provide treatments to the patients in the early stage. It is important to block or delay the development of dementia.

## Limitation

The limitations of this study are as follows. First, the sample size of the current study may be relatively small, and future studies with larger sample sizes are required. Second, because our sample size was small, we could not group different ages, the degree of cognitive impairments, and drug treatments separately. In this study, we attempted to select patients with type 2 diabetes who are below 70 years, and the average age of our patients in this study was 48.78. However, the small sample size was unsuitable for further grouping. Further studies with larger samples can consider age grouping and have stricter control of drug use, which may make the model more objective. Third, brain structural images were used to construct a classification model. However, previous studies have shown that functional brain changes occur earlier than structural changes. Therefore, future studies using brain function images or multimodal MRI images of patients with T2DM to construct a classification model are necessary.

## Data Availability Statement

The raw data supporting the conclusions of this article will be made available by the authors, without undue reservation.

## Ethics Statement

The studies involving human participants were reviewed and approved by the Ethics Committee of Guangzhou University of Traditional Chinese Medicine. The patients/participants provided their written informed consent to participate in this study.

## Author Contributions

JW, XM, SK, XY, YR, and HH collected the data. XT and SQ contributed to the study design, are the guarantors of this manuscript, had full access to all the data in the study, and took responsibility for the integrity of the data and the accuracy of the data analysis. XT wrote this manuscript. CQ, ML, YL, YC, WL, and YL performed the analysis. All authors contributed to the article and read and approved the final manuscript.

## Funding

This work was supported by the grants from the Key International Cooperation Project of National Natural Science Foundation of China (81920108019) and the Medical Scientific Research Foundation of Guangdong Province (A2021182).

## Conflict of Interest

The authors declare that the research was conducted in the absence of any commercial or financial relationships that could be construed as a potential conflict of interest. The reviewer FL declared a past co-authorship with the author SQ to the handling editor.

## Publisher's Note

All claims expressed in this article are solely those of the authors and do not necessarily represent those of their affiliated organizations, or those of the publisher, the editors and the reviewers. Any product that may be evaluated in this article, or claim that may be made by its manufacturer, is not guaranteed or endorsed by the publisher.
