# Supplementary material for: Convolutional Neural Networks for Classification of T2DM Cognitive Impairment Based on Whole Brain Structural Features
Source: Front Neurosci. 2022 Jul 19;16:926486. doi: 10.3389/fnins.2022.926486 (PMC9344913; doi:10.3389/fnins.2022.926486)
Supplement: Supplementary file 1 [file Data_Sheet_1.PDF]

The information of 21 clinical features (training set)

subject	HbA1c	Fasting blood glucose	Fasting insulin	Systolic pressure	Triglycerides	Total cholestero	Low density lipoprotein	Fasting C-peptide	BMI	AVLT (immidiate)	AVLT (5min)	AVLT (delay)	AVLT (recall)	TMT-A (s)	TMT-B (s)	DST (order)	DST (reverse)	MMSE	Grooved pegboard (right) (s)	Grooved pegboard (left) (s)	Digit-symbol test
1	13.71	10.52	0.98	124	0.64	6.53	5.27	0.526	21.36	20	6	6	10	65	52	10	5	/	73	76	53
2	10.53	7.69	12.62	139	1.54	4.69	3.44	3.25	28.08	10	4	6	6	42	118	9	2	/	76	85	/
3	6.52	7.96	7.00	161	4.63	1.12	3.06	/	25.63	22	9	7	10	30	59	7	5	/	/	/	/
4	7.11	7.88	7.79	121	2.12	5.11	3.29	2.26	24.09	28	11	11	12	76	117	5	3	27	77	79	/
5	7.12	8.64	20.55	147	3.17	3.68	2.13	3.35	27.76	22	10	10	11	44	44	8	4	/	99	118	28
6	6.60	7.52	6.18	110	/	/	/	1.81	20.38	20	9	9	9	39	34	9	6	/	84	79	59
7	8.23	8.50	4.68	156	2.16	4.62	3.03	1.61	21.21	23	9	9	12	77	46	7	4	/	75	78	31
8	9.60	7.32	/	158	0.74	3.99	2.57	1.43	27.34	21	7	6	11	103	67	9	4	/	80	90	41
9	9.12	8.02	/	144	1.18	5.37	2.81	/	26.29	/	/	/	/	/	/	7	/	/	109	125	/
10	10.35	7.52	3.41	134	2.01	6.82	5.60	0.41	24.17	21	8	7	9	34	27	7	5	29	59	73	44
11	12.91	11.11	3.92	134	7.31	8.05	2.52	1.27	23.45	26	11	11	12	49	49	9	8	/	93	100	53
12	13.17	7.73	5.19	138	0.81	4.39	3.21	1.75	/	18	7	7	12	50	41	8	5	/	91	80	49
13	7.52	8.02	4.01	125	1.58	5.04	3.43	1.73	25.60	20	7	8	9	52	43	5	5	/	72	78	42
14	9.65	7.66	7.15	131	3.77	4.58	2.62	/	22.77	24	10	9	12	37	30	7	4	/	90	110	51
15	6.63	8.91	16.6	123	2.41	4.69	3.16	3.73	27.91	32	12	12	12	38	28	12	10	/	70	75	60
16	11.01	7.93	4.05	125	0.74	4.13	2.91	1.14	21.31	26	8	8	12	33	36	8	6	/	62	85	61
17	8.43	9.54	4.69	126	4.02	3.69	4.26	/	25.43	16	10	9	12	49	58	8	5	/	73	75	33
18	13.83	13.21	11.30	127	0.99	3.37	2.34	0.76	20.24	21	9	9	8	39	33	7	4	/	77	94	41
19	8.64	8.96	11.32	172	2.79	6.67	/	/	25.06	25	10	11	12	43	35	7	5	/	/	/	/
20	7.71	8.15	/	115	0.73	5.36	4.12	/	22.83	23	9	7	12	38	30	8	4	/	99	93	42
21	4.90	7.02	7.21	115	0.96	3.15	1.47	1.22	21.23	24	9	8	12	44	116	10	4	/	64	73	/
22	8.82	8.86	12.03	120	4.34	5.73	3.43	3.33	27.63	24	8	9	12	21	33	8	8	/	63	70	73
23	8.54	7.01	49.11	139	1.16	4.38	2.72	2.51	32.11	20	8	8	8	46	25	8	4	/	84	87	49
24	11.72	11.91	5.04	104	1.46	3.45	2.22	1.69	22.15	29	11	12	12	35	22	10	8	/	61	59	56
25	11.71	12.64	13.12	124	7.68	4.76	2.13	4.47	26.93	28	12	12	12	37	36	5	5	30	74	78	60
26	7.10	7.65	13.14	144	1.54	6.34	4.91	/	23.52	29	11	12	12	36	29	5	5	/	81	83	47
27	8.12	7.79	13.42	148	1.79	4.10	2.76	3.00	25.39	26	10	10	12	27	24	5	3	30	67	94	58
28	12.94	7.07	2.06	142	2.24	5.43	3.93	0.54	21.33	27	11	11	12	35	49	8	4	26	78	80	43
29	6.41	7.38	5.17	136	1.42	3.84	2.60	/	25.39	25	12	12	12	50	52	7	4	/	103	97	50
30	6.36	7.11	8.88	138	0.71	5.68	4.25	1.75	21.22	28	11	10	12	43	53	8	5	29	56	66	54
31	11.31	13.67	16.91	124	7.92	5.99	2.49	/	26.29	27	11	7	5	45	42	8	7	/	57	71	40
32	10.86	11.82	10.22	143	2.26	3.14	1.93	2.56	18.14	25	11	11	12	61	41	11	7	30	70	69	58

subject	HbA1c	Fasting blood glucose	Fasting insulin	Systolic pressure	Triglycerides	Total cholestero	Low density lipoprotein	Fasting C-peptide	BMI	AVLT (immidiate)	AVLT (5min)	AVLT (delay)	AVLT (recall)	TMT-A (s)	TMT-B (s)	DST (order)	DST (reverse)	MMSE	Grooved pegboard (right) (s)	Grooved pegboard (left) (s)	Digit-symbol test
33	11.01	10.52	12.18	122	1.62	5.12	3.85	2.29	29.75	23	8	8	12	70	68	8	6	24	60	77	47
34	10.82	7.88	4.61	142	2.67	5.53	3.97	1.57	23.38	28	12	12	12	34	27	9	4	29	65	81	54
35	13.04	7.99	1.91	127	2.32	7.77	5.89	1.13	27.16	28	12	11	12	29	26	9	5	/	65	76	52
36	8.43	9.95	25.95	128	1.27	4.37	3.25	4.32	25.71	23	10	10	12	37	32	6	5	30	76	83	50
37	9.48	7.55	6.75	131	3.85	6.61	4.62	1.8	26.52	22	7	9	12	48	42	9	4	/	69	68	46
38	7.47	10.50	9.52	128	1.94	6.32	4.84	/	23.67	25	12	/	/	42	28	8	6	30	70	72	64
39	9.12	7.81	6.28	126	1.63	4.25	3.00	1.71	26.71	26	12	11	12	63	50	8	4	/	82	90	31
40	9.60	8.15	18.9	131	7.61	2.97	5.81	/	28.89	18	9	10	12	82	44	9	6	/	/	/	/
41	9.01	10.30	/	140	1.38	4.58	2.89	/	22.80	24	7	7	12	50	31	8	3	/	110	110	/
42	9.41	8.57	6.87	141	1.43	5.87	4.38	1.75	21.25	12	5	5	12	66	65	8	4	/	117	100	/
43	11.84	7.77	2.50	117	1.97	4.62	3.16	1.18	24.81	25	11	11	12	46	45	8	5	/	77	86	41
44	6.82	7.21	13.69	124	1.39	5.85	4.11	2.71	26.67	22	11	11	12	54	30	5	5	/	77	76	51
45	10.93	9.07	8.58	127	0.87	4.20	2.75	2.36	25.41	10	4	2	10	62	/	4	2	/	121	123	/
46	8.93	10.59	/	132	/	/	/	/	23.15	17	7	10	10	48	48	7	4	/	125	114	41
47	10.01	17.50	/	135	1.25	4.32	2.47	/	26.44	19	8	7	10	51	55	8	3	/	97	90	23
48	6.61	8.51	17.47	134	1.97	4.21	2.84	2.92	26.07	20	6	6	11	46	46	9	6	/	100	92	28
49	11.51	9.04	5.40	126	1.03	3.88	2.74	1.64	20.55	25	9	8	11	70	95	10	2	/	88	80	39
50	6.82	9.41	2.76	134	0.83	3.48	1.55	1.10	26.95	/	/	/	/	/	/	/	/	/	138	131	/
51	10.21	11.75	3.93	142	0.71	5.49	3.82	1.52	19.03	11	3	3	4	54	106	7	5	/	231	201	/
52	6.60	8.60	/	119	/	/	/	/	27.24	18	6	7	7	70	82	9	2	/	181	182	33
53	8.44	7.55	16.97	121	1.62	3.47	2.30	3.25	24.61	19	6	5	9	75	118	6	2	/	73	75	/
54	12.56	7.19	/	132	2.72	5.38	3.91	2.97	22.86	12	5	4	8	110	33	6	4	/	70	80	36
55	7.07	7.56	/	128	/	/	/	/	20.97	17	7	6	6	57	38	8	3	/	88	98	37
56	11.25	11.26	/	126	/	/	/	/	26.73	19	6	5	9	53	45	9	4	/	83	107	50
57	9.83	10.24	8.22	125	0.99	3.61	2.58	/	23.00	22	7	12	12	185	75	9	4	/	/	/	/
58	8.06	7.26	17.16	138	1.51	4.51	2.96	3.21	24.83	23	4	6	12	122	52	10	6	/	65	74	30
59	11.13	7.88	3.69	123	1.20	5.25	3.95	1.49	25.60	25	8	9	12	62	52	8	7	25	69	90	46
60	7.45	7.32	/	146	2.07	5.13	3.58	/	21.48	19	8	8	12	105	76	8	4	/	114	175	9
61	12.10	11.12	24.11	148	1.80	3.22	2.17	4.64	26.72	/	/	/	/	107	86	/	/	/	105	119	/
62	11.21	7.92	1.36	145	1.30	4.37	3.18	0.72	27.68	25	11	11	12	35	27	7	5	27	68	67	42
63	12.21	10.49	7.74	132	2.03	4.09	2.76	2.32	22.46	14	5	6	/	86	73	9	4	/	115	104	37
64	6.61	8.89	11.88	129	1.99	4.77	3.11	3.93	26.02	24	10	9	12	41	32	5	3	/	83	78	33
65	7.93	7.35	2.65	116	1.21	5.10	3.87	1.07	23.74	25	10	7	12	61	50	8	4	/	106	111	54

subject	HbA1c	Fasting blood glucose	Fasting insulin	Systolic pressure	Triglycerides	Total cholestero	Low density lipoprotein	Fasting C-peptide	BMI	AVLT (immidiate)	AVLT (5min)	AVLT (delay)	AVLT (recall)	TMT-A (s)	TMT-B (s)	DST (order)	DST (reverse)	MMSE	Grooved pegboard (right) (s)	Grooved pegboard (left) (s)	Digit-symbol test
66	12.73	8.79	18.84	146	0.74	4.95	3.8	0.34	21.29	26	10	7	11	93	56	9	7	/	116	112	25
67	11.52	11.8	5.09	105	1.25	4.75	3.18	1.78	18.68	11	0	0	0	76	77	/	/	/	/	/	/
68	6.80	8.96	19.12	126	4.57	5.54	2.92	3.68	23.51	33	12	12	12	45	49	8	7	/	90	95	95
69	9.25	7.53	6.48	129	3.34	3.30	1.69	1.99	28.25	25	10	10	12	67	57	5	2	/	79	81	37
70	11.22	8.53	2.07	154	3.68	5.34	2.99	1.11	23.42	14	2	5	/	63	63	9	3	/	/	/	/
71	8.63	11.16	10.92	134	1.25	3.69	2.49	/	27.21	20	9	9	12	32	28	8	6	/	77	76	52
72	11.50	15.90	8.82	122	2.36	/	/	1.83	24.57	24	9	10	12	20	23	9	4	29	64	98	44
73	5.71	7.87	38.11	133	4.02	4.21	2.36	2.65	19.03	17	7	7	7	/	/	9	5	25	65	74	60
74	12.26	12.08	20.79	127	1.72	5.13	4.08	1.51	21.87	22	7	7	5	42	38	9	6	/	65	99	57

The information of 21 clinical features (test set)

subject	HbA1c	Fasting blood glucose	Fasting insulin	Systolic pressure	Triglycerides	Total cholestero	Low density lipoprotein	Fasting C-peptide	BMI	AVLT (immidiate)	AVLT (5min)	AVLT (delay)	AVLT (recall)	TMT-A (s)	TMT-B (s)	DST (order)	DST (reverse)	MMSE	Grooved pegboard (right) (s)	Grooved pegboard (left) (s)	Digit-symbol test
1	10.10	8.89	1.76	119	/	/	/	0.72	21.60	29	12	12	12	48	26	6	4	/	91	108	51
2	7.12	7.23	9.71	144	0.96	3.54	2.63	2.39	21.78	26	9	9	12	75	43	6	4	/	99	89	33
3	8.22	7.94	7.09	131	1.50	4.25	2.83	1.56	21.01	26	11	11	12	27	19	9	7	/	78	70	56
4	11.36	9.34	83.08	142	2.12	5.86	4.58	3.92	23.11	24	10	10	12	58	50	8	3	/	104	114	24
5	12.23	8.9	3.82	125	1.61	3.57	2.08	1.27	22.66	24	10	8	12	37	32	10	5	/	75	74	47
6	9.61	8.33	5.34	130	1.66	5.00	3.84	1.49	24.44	19	8	8	12	65	30	8	4	/	63	75	52
7	8.54	9.23	/	123	/	/	/	/	27.34	18	9	10	10	45	39	6	3	/	82	99	36
8	6.91	7.62	6.88	119	0.65	3.58	2.59	1.86	27.34	22	8	9	12	46	33	6	3	/	70	76	/
9	6.65	7.66	0.79	127	0.70	4.33	2.74	0.41	23.60	29	10	9	12	54	39	8	3	/	82	91	50
10	6.60	7.27	/	117	0.98	3.46	1.79	/	26.21	20	6	6	12	/	/	10	7	/	93	84	/
11	7.36	7.91	10.25	134	1.87	4.68	3.09	/	22.49	26	11	12	12	28	20	9	6	/	/	/	/
12	7.14	8.86	8.12	144	4.31	5.03	2.97	2.28	23.72	20	10	10	12	45	38	5	4	30	66	68	37
13	15.13	8.72	3.62	126	0.61	3.59	1.81	0.97	16.62	28	11	12	12	39	38	8	4	/	56	67	55
14	8.06	7.96	19.91	128	1.54	4.96	3.67	/	27.11	20	9	9	12	29	29	7	5	28	58	74	63
15	6.92	9.04	28.32	151	3.48	4.88	3.03	4.16	27.73	24	8	8	11	29	25	9	5	29	60	65	57
16	6.94	7.03	9.57	143	1.02	3.61	2.59	/	23.58	23	10	10	12	45	34	8	5	29	75	78	59
17	7.67	7.52	48.03	140	2.51	4.57	3.17	2.48	20.32	31	12	12	12	37	32	5	5	29	69	75	46
18	9.71	9.40	3.53	120	2.45	4.09	2.56	1.72	26.03	19	5	6	4	84	53	8	2	/	146	124	17
19	8.60	12.89	/	124	1.42	3.53	2.79	1.09	21.48	12	6	3	6	110	104	9	3	/	133	107	/
20	8.34	7.09	/	134	2.17	6.79	1.09	/	25.84	22	8	7	10	136	66	6	2	/	123	143	7
21	9.76	9.87	/	123	/	/	/	/	26.16	18	0	0	10	105	60	8	3	/	93	102	33
22	11.32	7.52	7.23	122	2.36	4.87	5.01	/	29.07	11	4	4	11	31	64	9	5	/	68	90	54
23	7.96	8,01	6.58	159	3.35	4.63	3.10	/	24.50	18	6	12	12	132	72	7	3	/	/	/	/
24	12.31	9.10	6.21	133	2.32	4.49	3.04	1.89	24.38	25	9	10	12	89	54	9	4	/	84	96	34
25	7.32	7.74	/	145	1.12	6.20	4.44	/	26.83	/	/	/	/	/	/	/	/	/	/	/	/
26	7.11	7.62	5.52	137	2.37	3.72	2.30	1.96	23.51	17	5	4	10	90	42	6	4	/	70	81	40
27	6.52	7.23	/	131	1.84	6.42	4.87	/	22.21	28	11	11	12	35	28	6	4	30	65	65	29
28	19.54	14.35	5.66	139	1.42	4.90	3.63	1.47	26.26	13	5	1	0	113	57	5	4	25	100	125	22
29	10.17	8.42	4.44	122	3.44	4.86	3.38	1.56	20.95	22	4	5	12	51	48	9	4	/	86	73	57
30	14.12	13.16	5.27	151	1.40	3.84	2.57	2.51	21.25	30	12	12	12	69	48	8	5	29	103	128	38
31	8.60	8.36	2.60	134	0.78	4.14	2.55	/	21.30	22	9	6	12	38	30	9	6	/	76	75	50
32	8.42	9.04	10.76	142	2.11	5.39	4.04	/	24.91	22	8	/	/	79	70	7	3	30	68	91	49

subject	HbA1c	Fasting blood glucose	Fasting insulin	Systolic pressure	Triglycerides	Total cholestero	Low density lipoprotein	Fasting C-peptide	BMI	AVLT (immidiate)	AVLT (5min)	AVLT (delay)	AVLT (recall)	TMT-A (s)	TMT-B (s)	DST (order)	DST (reverse)	MMSE	Grooved pegboard (right) (s)	Grooved pegboard (left) (s)	Digit-symbol test
33	6.73	7.19	9.90	156	0.99	3.96	2.26	2.77	24.84	15	7	5	0	73	51	7	3	30	128	135	23

Note:

1. The number of subjects was the rearranged number after randomly grouping, not the original number.

2. ‘/’ represented the missing data.

3. The data of the cognitive scales represented scores except the ‘Grooved pegboard’, and the data of the TMT and grooved pegboard test represented the time the patient needed to complete the task, which were calculated in seconds.
